# Bilateral Recurrent Laryngeal Nerve Paralysis Manifesting as Long COVID

**DOI:** 10.7759/cureus.27792

**Published:** 2022-08-08

**Authors:** Hiroshi Okuda, Chikako Kunieda, Hirofumi Shibata, Toshimitsu Ohashi, Takenori Ogawa

**Affiliations:** 1 Otolaryngology - Head and Neck Surgery, Gifu University Graduate School of Medicine, Gifu, JPN; 2 Otolaryngology - Head and Neck Surgery, Hashima Municipal Hospital, Hashima, JPN

**Keywords:** coronavirus disease 2019, clinical case report, otolaryngology-head and neck surgery, long covid syndrome, airway obstruction, recurrent laryngeal nerve palsy, bilateral vocal cord paralysis, covid-19

## Abstract

Management with ventilation is used for severe cases of coronavirus disease 2019 (COVID-19). After extubation, recurrent laryngeal nerve paralysis due to various factors may occur. Almost all cases of paralysis develop unilaterally; however, bilateral recurrent laryngeal nerve paralysis occurs rarely. Such cases may be fatal due to upper air obstruction, and patients are forced to adhere to restrictions after a tracheotomy. The present case illustrates bilateral recurrent laryngeal nerve paralysis that occurred 48 hours after withdrawal from the ventilator. A 75-year-old woman with a history of hypertension came to our hospital with a history of fever and cough for five days. She was diagnosed with pneumonia due to COVID-19 via polymerase chain reaction using her saliva, and ground-glass opacity was found in both lung fields on chest X-ray and computed tomography (CT). Mechanical ventilation, steroids, remdesivir, and baricitinib were administered. The patient's fever and oxygenation status improved with these treatments, and she was weaned from the ventilator on the eighth day of hospitalization. She had no symptoms immediately. However, 48 hours after extubation, bilateral recurrent laryngeal nerve paralysis was suspected. Thus, oral intubation was immediately introduced and a tracheostomy was performed. Vocal cord movement disorders continued for eight weeks, and during that period, the patient displayed hoarseness and suffered from dysphagia. We considered that nerve disorders from severe acute respiratory syndrome coronavirus 2 (SARS-CoV-2), in addition to the compression by the endotracheal tube, caused bilateral recurrent laryngeal nerve paralysis. The neural injury by SARS-CoV-2 may prolong and manifest as "Long COVID."

## Introduction

The global pandemic of coronavirus disease 2019 (COVID-19) is ongoing, and there is no expectation that it will end. Various mutant strains have appeared, but the infection route is commonly via respiratory droplets during close face-to-face contact [1]. The causative virus of COVID-19, severe acute respiratory syndrome coronavirus 2 (SARS-CoV-2), invades mainly through the nasal mucosa and respiratory epithelium and causes local inflammation [2,3]. On the other hand, just as represented by direct damage to the olfactory nerve via the nasal mucosa, viral neuropathy due to SARS-CoV-2 is known as well [2].

Ventilation management is performed in severe cases of COVID-19. The well-known complications associated with ventilatory management include recurrent laryngeal nerve palsy. This paralysis is primarily caused by injury due to the pressure through compression of the intubation tube or extension of the neck. This complication is usually unilateral, occurs immediately after extubation, and improves relatively early [4]. There are few reports describing bilateral and prolonged paralysis of recurrent laryngeal nerve. Here, we report a rare case of bilateral recurrent laryngeal nerve palsy that occurred shortly after ventilator withdrawal and took a long time to improve.

## Case presentation

A 75-year-old woman with a history of hypertension came to our hospital with a history of fever and cough for five days. COVID-19 was detected via polymerase chain reaction (PCR) using the patient’s saliva, and ground-glass opacity was found in both lung fields on chest X-ray and computed tomography (CT). The patient was thus diagnosed with pneumonia due to COVID-19.

Oxygen inhalation (started at 4 l/min and increased to 10 l/min with mask oxygenation) and intravenous administration of dexamethasone (started at 6.6 mg daily and tapered steadily to off for 10 days), remdesivir (200 mg daily for eight days), and baricitinib (2 mg daily for seven days) were administered. On the second day of hospitalization, oxygen saturation decreased, and the patient was placed on mechanical ventilation.

Because the patient's fever and oxygenation status improved with these treatments, she was weaned from the ventilator on the eighth day of hospitalization. She had no symptoms immediately, but 48 hours after extubation, she had dyspnea and decreased oxygen saturation.

**Figure 1:**
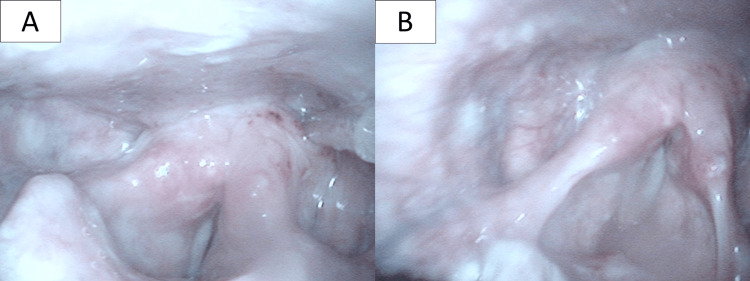
Laryngeal findings 48 hours after extubation The vocal cords on both sides were fixed in the midline during both inspiration and exhalation, and the glottic space was slit-shaped. (A) Inspiration phase. (B) Exhalation phase.

Laryngeal examination revealed both sides of the vocal cords to be fixed in the midline during both inspiration and expiration phases, and the glottic space was slit-shaped (Figure 1).

Oral intubation was immediately introduced and tracheostomy was performed in a COVID-19-compliant operating room.

**Figure 2:**
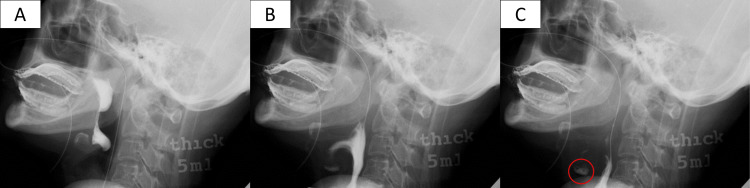
Swallowing videofluorography (A-C) Findings in chronological order. Because of delayed laryngeal elevation, water swallowed accumulates in the hypopharynx (A); part of it invades the larynx (B) and crosses the glottis into the trachea (C). Circle: water aspirated.

Postoperative laryngeal examination showed that her vocal cords were still fixed in the paramedian position. In addition, marked saliva retention was observed in the larynx. A swallowing videofluorography revealed that swallowed water invaded the larynx because of a delay in laryngeal elevation and a decrease in pharyngeal contractility (Figure 2).

**Figure 3:**
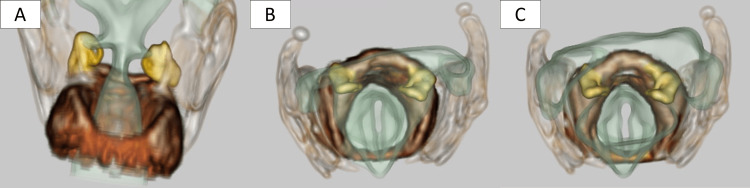
Laryngeal three-dimensional CT scan Arytenoid cartilage does not show any deviation suggestive of dislocation. There is almost no change in the position of the vocal cords during either the inspiration or the vocalization phase, indicating that their movement is highly impaired. (A, B) Inspiration phase. (C) Vocalization phase. Yellow: arytenoid cartilage; dark brown: cricoid cartilage; light brown: thyroid cartilage; light green, V-shaped structure at ventral side: vocal cord.

Laryngeal three-dimensional CT scan imaging revealed no sign of dislocation of the arytenoid cartilage. Furthermore, almost no movement of the vocal cord was observed during inspiration and vocalization (Figure 3).

In addition, no other causative findings of vocal cord movement disorders such as inflammatory scar and tumor of the larynx were observed. Thus, bilateral recurrent laryngeal nerve paralysis was suspected. Various examinations were performed to determine the cause of nerve paralysis. Magnetic resonance imaging of the head and CT of the neck and chest revealed no tumor or abscess. Virus antibody titer test for herpes zoster virus was negative.

The aforementioned examination results indicated the possibility of bilateral recurrent laryngeal nerve paralysis due to endotracheal tube placement and/or COVID-19 neuropathy.

The patient’s fever decreased with the administration of antibiotics (sulperazone/tazobactam 13.5 g daily for seven days, followed by cefmetazole 2 g daily for seven days). Oral vitamin B12 1500 mg daily was administered. Rehabilitation of swallowing was started while on tube feeding.

**Figure 4:**
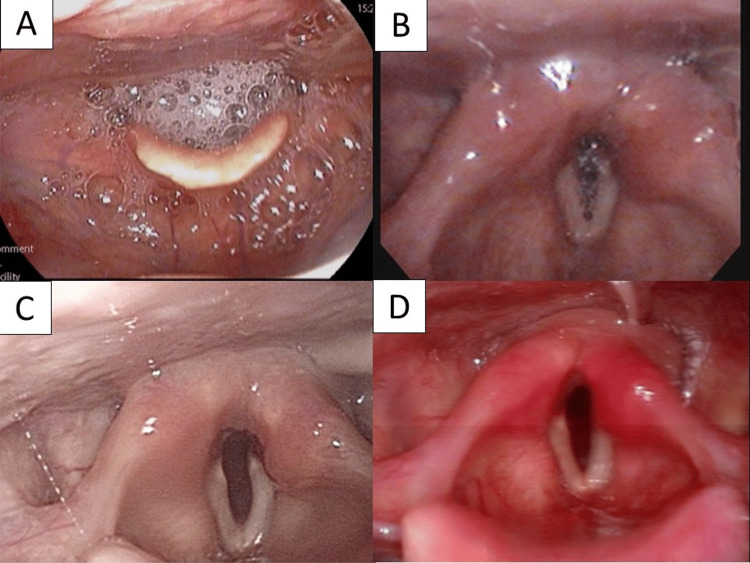
Progression of laryngeal findings Vocal cord dilation gradually improved starting on the right side. Saliva retention in the larynx gradually decreased. (A) Two weeks, (B) four weeks, (C) six weeks, and (D) eight weeks after tracheostomy. Image D appears to be congested because the laryngeal fiberscope is different.

Vocal cord movement disorders were prolonged. The disorder on the left side of the vocal cord was prolonged compared with that on the right side, but the patient's hoarseness gradually improved five weeks after surgery. Regarding swallowing function, oral intake was resumed three weeks after surgery, and tube feeding was discontinued five weeks after surgery. Seven weeks after surgery, the tracheostomy was decannulated and eight weeks after surgery, the tracheal stoma was closed naturally and the patient was discharged (Figure 4).

## Discussion

Causes of vocal cord motion disorder are diverse, such as scarring, iatrogenic causes, malignancy, central nervous system pathology, and systemic diseases, but may also be idiopathic [5]. With regard to artificial respiration, one of the causes of vocal cord motion disorder after withdrawal from a ventilator is palsy of the recurrent laryngeal nerve or the inferior laryngeal nerve due to compression by the endotracheal tube or from cervical extension [6-8]. Intubation lasting over six hours may cause nerve damage [9]. In addition, glottic insufficiency results in reduced swallowing pressure and can cause dysphagia. Symptoms of hoarseness and dysphagia may appear 30 minutes to 36 hours after extubation [10]. Early resolution of this paralysis has been reported, in some cases, within a week [10].

Another mechanism of nerve paralysis is a viral infection. Two major pathways have been proposed for the invasion of the virus into the central nervous system: the hematogenous pathway via the blood-brain barrier and the retrograde pathway from the peripheral nerves [11]. With regard to the latter, the currently hypothesized pathomechanism is that the virus is activated when transmembrane protease serine 2 (TMPRSS2) binds to the angiotensin-converting enzyme 2 receptor (ACE2-R) and enters the nervous system from the epithelium cells [12]. It is known that ACE2-R and TMPRSS2 are co-expressed in the nasal mucosal epithelium and the pulmonary respiratory epithelium [2,3]. On the other hand, similar findings have been confirmed in the laryngeal mucosal epithelium in rats, suggesting that SARS-CoV-2 may be transmitted from the laryngeal mucosa and cause local damage in humans [13]. Moreover, patients with severe illnesses, such as pneumonia, are most likely to develop neurological symptoms [14,15].

There are very few reports of bilateral recurrent laryngeal nerve palsy during the course of COVID-19 [12,16], with similar findings that paralytic symptoms develop a little later than ventilator withdrawal and do not improve within a short period of time. Previous reports could not ascertain whether the cause of nerve palsy was viral or mechanical in origin; however, in one publication, the authors proved that the recurrent laryngeal nerve did not work using electromyography [17].

In the present report, hoarseness occurred 24 hours after extubation, making it likely that neuropathy due to nerve compression had occurred. However, in this case, bilateral vocal cord motion disorder occurred 48 hours after extubation. Furthermore, the symptoms of paralysis continued for nearly eight weeks. Therefore, we believe that there is a possibility that the viral infection due to COVID-19 caused nerve paralysis, in addition to nerve compression.

Ongoing symptomatic COVID-19 is defined as signs and symptoms that persist between four and 12 weeks from the onset of the infection. Post-COVID-19 syndrome is defined as the persistence of symptoms beyond 12 weeks from the date of onset. The term "Long COVID" includes both ongoing symptomatic COVID-19 and post-COVID-19 syndrome [18]. Common symptoms in people with "Long COVID" are profound fatigue, respiratory symptoms such as breathlessness and cough, gastrointestinal symptoms, rashes or hair loss, and neurocognitive issues, including memory and concentration problems [18,19]. Moreover, severe respiratory injury and extreme mechanical ventilation can result in neuropathies [19]. We suggest that prolonged recurrent laryngeal nerve paralysis should be considered a symptom of "Long COVID." If the paralysis occurs bilaterally, the patient’s quality of life is severely affected. Therefore, clinicians should consider the long-term effects after infection with SARS-CoV-2.

## Conclusions

Over the course of severe COVID-19, multiple factors may combine to cause the development of bilateral vocal cord motion disorder. When symptoms such as hoarseness and dysphagia appear, even if not immediately after extubation, bilateral recurrent laryngeal nerve paralysis may occur. It is necessary to secure the airway quickly and concern the possibility of long-term laryngeal dysfunction as "Long COVID".
